# Treatment with Obestatin—A Ghrelin Gene-Encoded Peptide—Reduces the Severity of Experimental Colitis Evoked by Trinitrobenzene Sulfonic Acid

**DOI:** 10.3390/ijms19061643

**Published:** 2018-06-01

**Authors:** Katarzyna Konarska, Jakub Cieszkowski, Zygmunt Warzecha, Piotr Ceranowicz, Anna Chmura, Beata Kuśnierz-Cabala, Krystyna Gałązka, Paweł Kowalczyk, Andrzej Miskiewicz, Thomas Jan Konturek, Michał Pędziwiatr, Artur Dembiński

**Affiliations:** 1 Department of Physiology, Faculty of Medicine, Jagiellonian University Medical College, 31-531 Cracow, Poland; kasia.konarska@uj.edu.pl (K.K.); jakub.cieszkowski@uj.edu.pl (J.C.); mpwarzec@cyf-kr.edu.pl (Z.W.); anna.1.chmura@uj.edu.pl (A.C.); artur.dembinski@uj.edu.pl (A.D.); 2 Department of Clinical Biochemistry, Faculty of Medicine, Jagiellonian University Medical College, 31-501 Cracow, Poland; beata.kusnierz-cabala@uj.edu.pl; 3 Department of Pathomorphology, Faculty of Medicine, Jagiellonian University Medical College, 31-531 Cracow, Poland; krystyna.galazka@uj.edu.pl; 4 Department of Animal Nutrition, The Kielanowski Institute of Animal Physiology and Nutrition, Polish Academy of Sciences, 05-110 Jablonna, Poland; p.kowalczyk@ifzz.pl; 5 Department of Periodontology and Oral Diseases, Medical University of Warsaw, 00-246 Warsaw, Poland; andrzej.miskiewicz@wum.edu.pl; 6 Department of Medicine, St. Elizabeth’s Medical Center, Tufts University School of Medicine, Boston, MA 02135, USA; konturek@me.com; 7 Second Department of General Surgery, Faculty of Medicine, Jagiellonian University Medical College, 31-501 Cracow, Poland; michal.pedziwiatr@uj.edu.pl

**Keywords:** colitis, TNBS, ghrelin, obestatin, DNA synthesis, interleukin-1β, colonic blood flow, myeloperoxidase

## Abstract

Obestatin is a 23-amino acid peptide derived from proghrelin, a common prohormone for ghrelin and obestatin. Previous studies showed that obestatin exhibited some protective and therapeutic effects in the gut. The aim of our presented study was to examine the effect of treatment with obestatin on trinitrobenzene sulfonic acid (TNBS)-induced colitis. In rats anesthetized with ketamine, colitis was induced through intrarectal administration of 25 mg of 2,4,6-trinitrobenzene sulfonic acid (TNBS). Obestatin was administered intraperitoneally at doses of 4, 8, or 16 nmol/kg, twice per day for four consecutive days. The first dose of obestatin was given one day before the induction of colitis, and the last one was given two days after administration of TNBS. Fourteen days after the induction of colitis, rats were anesthetized again with ketamine, and the severity of colitis was determined. The administration of obestatin had no effect on the parameters tested in rats without the induction of colitis. In rats with colitis, administration of obestatin at doses of 8 or 16 nmol/kg reduced the area of colonic damage, and improved mucosal blood flow in the colon. These effects were accompanied by a reduction in the colitis-evoked increase in the level of blood leukocytes, and mucosal concentration of pro-inflammatory interleukin-1β. Moreover, obestatin administered at doses of 8 or 16 nmol/kg reduced histological signs of colonic damage. The administration of obestatin at a dose of 4 nmol/kg failed to significantly affect the parameters tested. Overall, treatment with obestatin reduced the severity of TNBS-induced colitis in rats. This effect was associated with an improvement in mucosal blood flow in the colon, and a decrease in local and systemic inflammatory processes.

## 1. Introduction

Obestatin is a 23-amino acid peptide, discovered by Zhang et al. in 2005, and is described as a ghrelin-associated peptide, derived from the post-translational processing of the preproghrelin gene [1]. It was isolated from the rat stomach [1,2,3] and that organ was identified as the major source of circulating obestatin [1,2,4]. On the other hand, further investigations revealed little or no immunoreactivity in the stomach is observed in young animals in fetal and neonatal periods in life [5,6]. Apart from the stomach, obestatin expression was also found in other tissues, such as the endocrine pancreas, adipose tissue, the lung, liver, skeletal muscle, mammary glands, and the male reproductive system [7,8,9,10,11,12,13,14].

Initially obestatin was reported to activate the G-protein-coupled receptor, GPR39 [1,15], but later studies did not confirm that obestatin was a ligand of this receptor [7,16,17,18,19,20]. More recently Granata et al. described that obestatin may bind to the glucagon-like peptide 1 receptor (GLP-1R) in adipocytes and pancreatic beta cells [8,21]. However, the specific receptor for obestatin is still unknown. On the other hand, numerous studies showed that administration of obestatin exhibited biological effects in various tissues [8,15,22,23,24]. These data indicated that obestatin receptors must be present in these tissues. 

Previous studies indicated that obestatin potentially suppressed motility of the gastrointestinal tract [25,26,27], regulated the secretion of insulin [8,21,28,29,30,31], protected against ischemia-reperfusion injury in various organs [22,23,32], inhibited platelet aggregation [33], prevented H_2_O_2_-induced damage in RGC-5 cells [34], and had multiple other functions in digestive system [35,36,37]. Moreover, obestatin possibly reflected and affected some clinical syndromes [38,39,40,41,42,43,44,45].

Previous studies showed that ghrelin—an alternative product of post-translational processing of preproghrelin—exhibited a protective effect in gastrointestinal mucosa against damage caused by harmful factors [46,47,48,49,50], and potentially inhibited the development of acute pancreatitis [51,52,53]. Ghrelin also had a therapeutic effect in the gastrointestinal tract [54]. It accelerated the healing of oral [55,56], gastric [57], and duodenal [55,57] ulcers, and colonic inflammation [58,59,60,61,62]. Ghrelin also exhibited a therapeutic effect in animal models of acute pancreatitis [63,64,65,66]. 

In the case of obestatin, there are some reports showing protective and therapeutic effects in the gastrointestinal tract. Obestatin inhibited the development of cerulein- and ischemia-reperfusion-induced pancreatitis, as well as reduced its severity and accelerated recovery [67,68,69]. Granata et al. reported that obestatin promoted the survival of pancreatic islets [8]. Obestatin increased gastric mucosal blood flow and cell proliferation, leading to acceleration of healing of gastric ulcers [70]. The determination of ratio of the serum level of obestatin to ghrelin was proposed as a marker form monitoring the activity of inflammatory process in inflammatory bowel disease (IBD) [71,72]. Its level was significantly reduced in patients with celiac disease after one year of a gluten-free diet [73]. Previous studies showed that administration of obestatin inhibited the development of, and accelerated the healing of colitis induced by acetic acid [74] or dextran sodium sulfate (DSS) [75]. However, in both these models of colitis, mucosal damage was the result of a direct deleterious effect of chemicals used on previously healthy mucosa. These models of colitis allows the study of the protective and therapeutic effect of various factors, but the mechanism of colonic damage in acetic acid- or DSS-induced colitis only moderately corresponds to the pathophysiology of mucosal damage observed in IBD. 

Trinitrobenzene sulfonic acid (TNBS)-induced colitis leads to a transmural inflammation of colonic wall, that well-corresponds with morphological features observed in clinical IBD [76,77,78]. This form of experimental colitis is known and studied for at least two decades [78,79,80]. TNBS does not cause intestinal inflammation itself, but it is rather a result of a delayed hypersensitivity reaction. TNBS haptenizes colonic autologous/microbial proteins, making them immunogenic to the host’s immune system. In fact, TNBS-induced colitis resembles the hapten-induced model. The Th1 lymphocytes-mediated immune response involves various cytokines, including interleukin-12 (IL-12) and tumor necrosis factor alpha (TNFα) which serve as effector cytokines, leading to transmural infiltration and inflammation [81,82,83]. Therefore, this animal model of colitis was generally considered to be the most compatible with pathophysiological mechanism of IBD in humans [78]. For this reason, we used TNBS-induced colitis in our current study, and the aim of this study was to evaluate the effect of obestatin administration on the severity of trinitrobenzene sulfonic acid (TNBS)-induced colitis.

## 2. Results

**Figure 1:**
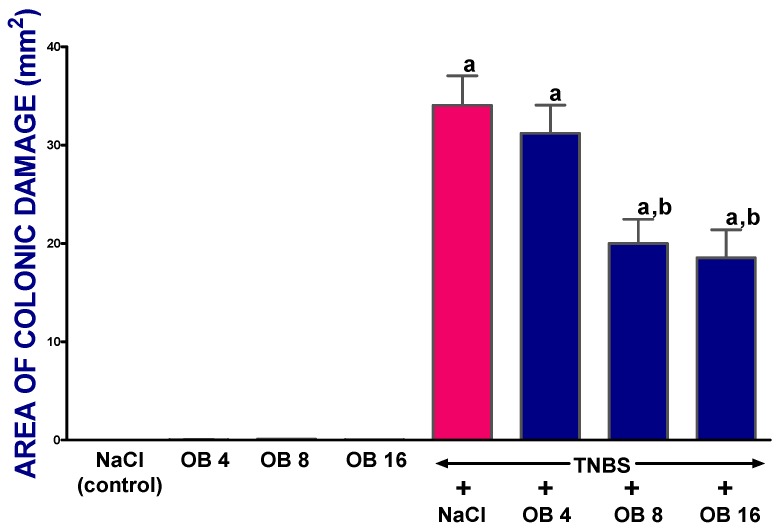
Effect of saline (NaCl) or obestatin given at doses of 4 nmol/kg (OB4), 8 nmol/kg (OB8), or 16 nmol/kg (OB16), and induction of trinitrobenzene sulfonic acid (TNBS)-induced colitis on the area of colon mucosal damage. Mean ± standard error of the mean (SEM). *n* = 10 in each group of animals. ^a^ *p* < 0.05 when compared with control; ^b^ *p* < 0.05 when compared with colitis + NaCl.

Figure 1 demonstrates the impact of obestatin on the area of colonic damage in TNBS-induced colitis. In saline- and obestatin-treated rats with no induced colitis, no mucosal damage was detected. In saline-treated rats, 14 days after colitis induction, the area of mucosal damage was 34.05 ± 3.01 mm^2^. Obestatin given at doses of 4, 8, and 16 nmol/kg diminished the damaged area by 9%, 41%, and 48%, respectively. The results for doses of 8 and 16 nmol/kg were statistically significant.

**Figure 2:**
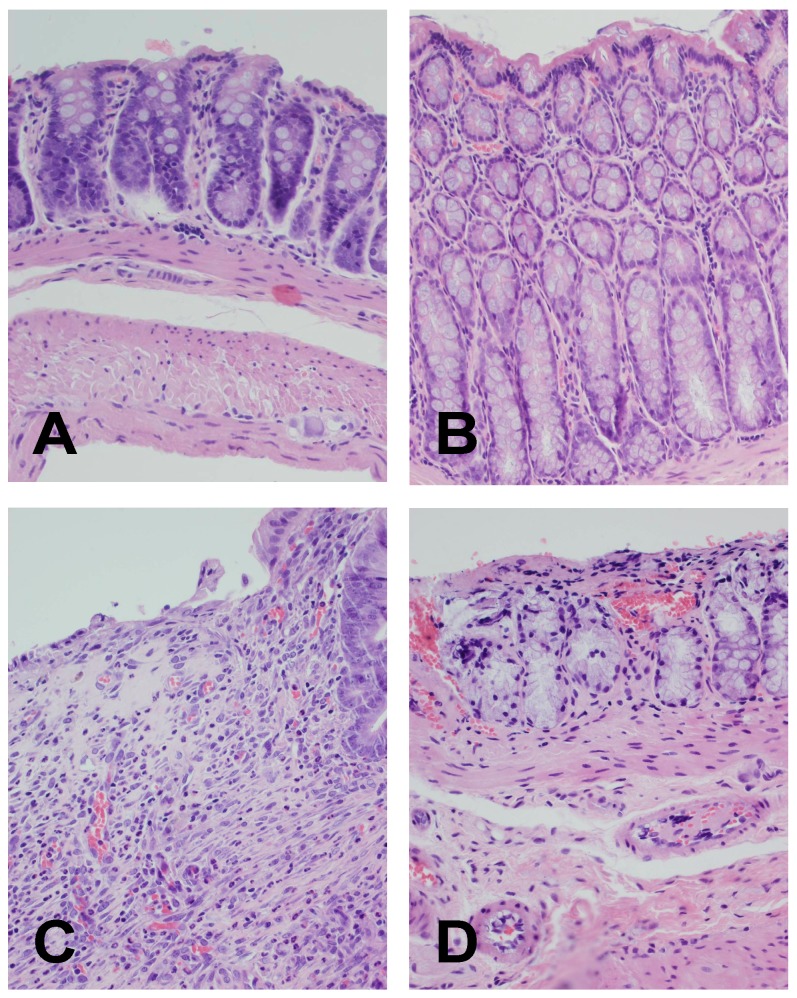
(**A**) Representative microscopic image of colonic mucosa observed in control rats without colitis; (**B**) Representative microscopic image of colonic mucosa observed 14 days after intraperitoneal (i.p.) treatment with obestatin at a dose of 8 nmol/kg without induction of colitis; (**C**) Representative microscopic image of colonic mucosa observed 14 days after induction of colitis evoked by TNBS; (**D**) Representative microscopic image of colonic mucosa observed 14 days after induction of colitis and i.p. treatment with obestatin at a dose of 8 nmol/kg. Hematoxylin and eosin stain. Original magnification 400×.

No damage was observed in microscopic images of mucosa in the control rats (Figure 2A,B, Table 1). After the period of 14 days, large lesions reaching the muscular membrane, associated with moderate or heavy inflammatory cell infiltration, and mild fibrosis were observed in colitis-induced rats (Figure 2C, Table 1). Obestatin treatment with doses of 4, 8, and 16 nmol/kg reduced the histological manifestation of colonic damage. Either small or no submucosal lesions were reported in rats treated with a dose of 8 nmol/kg, and small or moderate inflammatory infiltration, and mild fibrosis were present (Figure 2D, Table 1). 

**Figure 3:**
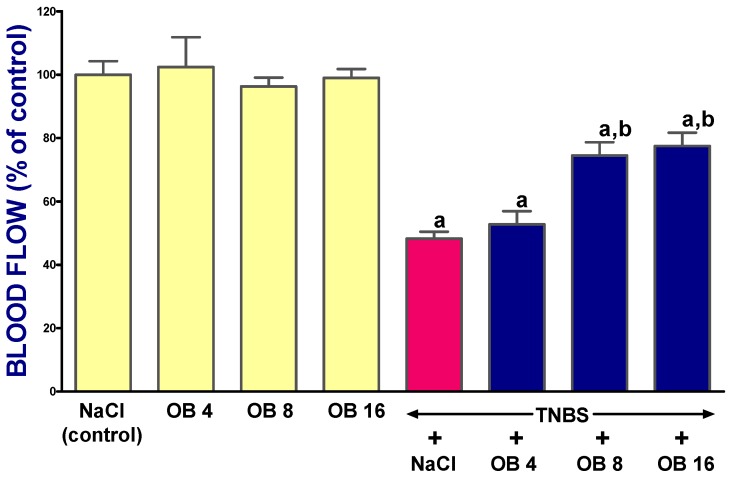
Effect of saline (NaCl) or obestatin given at doses of 4 nmol/kg (OB4), 8 nmol/kg (OB8), or 16 nmol/kg (OB16), and induction of TNBS-induced colitis on mucosal blood flow in the colon. Mean ± SEM. *n* = 10 in each group of animals. ^a^ *p* < 0.05 when compared with control; ^b^ *p* < 0.05 when compared with colitis + NaCl.

In rats without the induction of colitis, administration of obestatin at any dose failed to affect colonic mucosal blood flow (Figure 3). Induction of colitis significantly reduces mucosal blood flow in the colon by 51%, and this effect was partly reversed by treatment with obestatin. Obestatin given at doses of 8 or 16 nmol/kg exhibited similar and statistically significant effects on mucosal blood flow in rats with colitis. The effect of obestatin given at a dose of 4 nmol/kg was statistically insignificant in comparison with mucosal blood flow observed in rats treated with NaCl after the induction of colitis (Figure 3).

**Figure 4:**
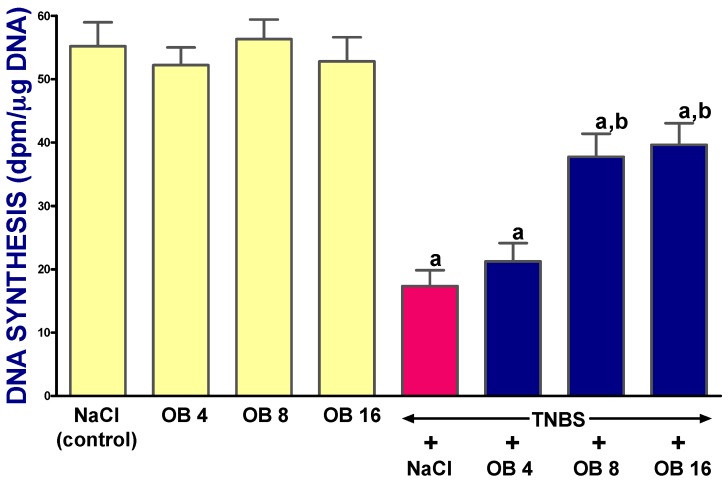
Effect of saline (NaCl) or obestatin given at doses of 4 nmol/kg (OB4), 8 nmol/kg (OB8), or 16 nmol/kg (OB16), and TNBS-induced colitis on mucosal DNA synthesis in the colon. Mean ± SEM. *n* = 10 in each group of animals. ^a^ *p* < 0.05 when compared with control; ^b^ *p* < 0.05 when compared with colitis + NaCl.

In control saline-treated animals without the induction of colitis, mucosal DNA synthesis in the colon reached a value of 55.2 ± 3.8 dpm/μg DNA (Figure 4). The administration of obestatin at doses of 4, 8, and 16 nmol/kg failed to affect DNA synthesis in colonic mucosa in rats without the induction of colitis. In saline-treated rats with TNBS-induced colitis, mucosal DNA synthesis in the colon was reduced to 33% of the control value. Treatment with obestatin partly reversed the colitis-evoked reduction in DNA synthesis in the colonic mucosa. This effect was statistically significant after obestatin administered at doses of 8 or 16 nmol/kg (Figure 4).

**Figure 5:**
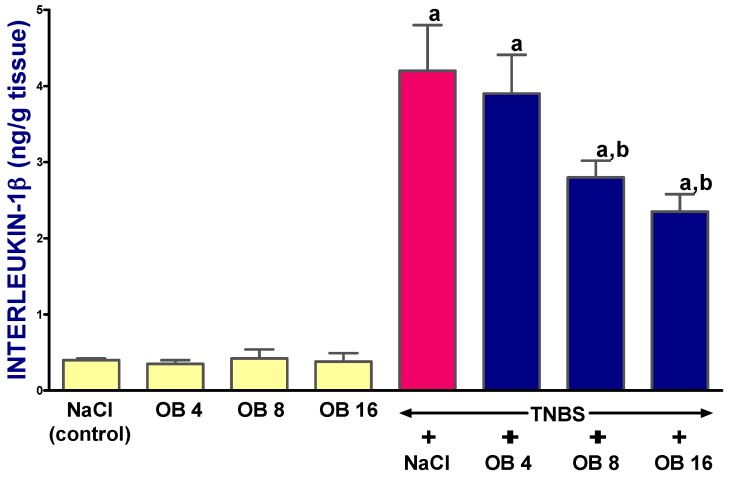
Effect of saline (NaCl) or obestatin given at doses of 4 nmol/kg (OB4), 8 nmol/kg (OB8), or 16 nmol/kg (OB16), and TNBS-induced colitis on mucosal concentration of interleukin-1β (IL-1β) in the colon. Mean ± SEM. *n* = 10 in each group of animals. ^a^ *p* < 0.05 when compared with control; ^b^ *p* < 0.05 when compared with colitis + NaCl.

In rats without colitis, administration of obestatin at any dose had no effect on the mucosal concentration of interleukin-1β (IL-1β) in the colon (Figure 5). The induction of colitis significantly increased the mucosal concentration of IL-1β in the colon. As shown in Figure 5, rats with colitis demonstrated more than a 10-fold increase in this parameter, 14 days after induction of this inflammation. The administration of obestatin at all doses used partly reversed the colitis-evoked increase in mucosal concentration of IL-1β, and this effect was statistically significant after obestatin administered at doses of 8 or 16 nmol/kg (Figure 5).

**Figure 6:**
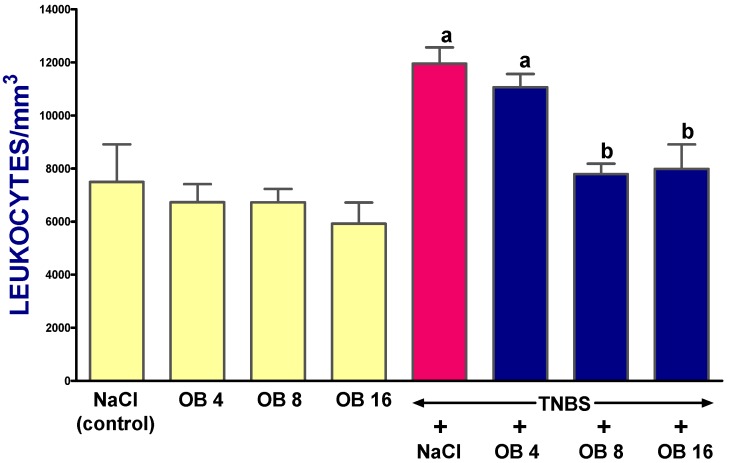
Effect of saline (NaCl) or obestatin given at doses of 4 nmol/kg (OB4), 8 nmol/kg (OB8), or 16 nmol/kg (OB16), and induction of TNBS-induced colitis on the number of blood leukocytes. Mean ± SEM. *n* = 10 in each group of animals. ^a^ *p* < 0.05 when compared with control; ^b^ *p* < 0.05 when compared with colitis + NaCl.

In control rats without colitis treated with saline, white-blood-cell (WBC) count reached a value of 7495 ± 1415 per 1 mm^3^ of blood (Figure 6). The administration of obestatin given at doses of 4, 8, and 16 nmol/kg had no significant effect on the number of leukocytes in 1 mm^3^ of blood in rats without TNBS-induced colitis (Figure 6). In rats treated with saline after the induction of colitis, almost a two-fold increase in the number of blood leukocytes was observed 14 days after the induction. Treatment with obestatin given at doses of 4, 8, and 16 nmol/kg reduced the colitis-evoked increase in the number of blood leukocytes. Obestatin administered at doses of 8 or 16 nmol/kg was statistically significant (Figure 6). 

**Figure 7:**
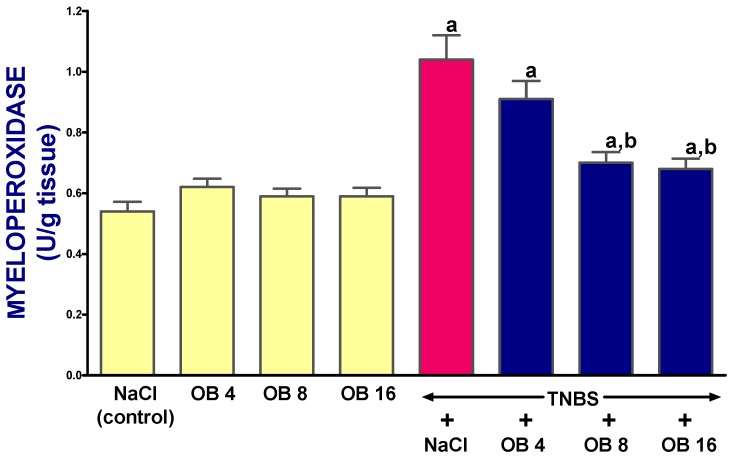
Effect of saline (NaCl) or obestatin given at doses of 4 nmol/kg (OB4), 8 nmol/kg (OB8), or 16 nmol/kg (OB16), and induction of TNBS-induced colitis on myeloperoxidase activity in colonic mucosa. Mean ± SEM. *n* = 10 in each group of animals. ^a^ *p* < 0.05 when compared with control; ^b^ *p* < 0.05 when compared with colitis + NaCl.

Obestatin given at doses of 4, 8, and 16 nmol/kg had no effect on mucosal myeloperoxidase (MPO) activity in the colon in rats without TNBS-induced colitis (Figure 7). In rats treated with saline after the induction of colitis, myeloperoxidase activity in colonic mucosa was increased almost two-fold 14 days after the induction. Treatment with obestatin given at doses of 4, 8, or 16 nmol/kg reduced the colitis-evoked increase in myeloperoxidase activity in the colonic mucosa. This effect was statistically significant after obestatin administered at doses of 8 or 16 nmol/kg (Figure 7).

## 3. Discussion

In this study, we investigated the effect of obestatin administration on the severity of trinitrobenzene sulfonic acid (TNBS)-induced colitis. We found that intraperitoneal administration of obestatin given at doses of 8 or 16 nmol/kg reduced the area of colonic damage, and improved mucosal blood flow in the colon. The administration of obestatin at a dose of 4 nmol/kg failed to significantly affect the parameters tested. This observation was in agreement with previous reports that obestatin at doses of 8 or 16 nmol/kg showed preventive and therapeutic effects on acute pancreatitis [67,68,69], chronic gastric ulcers [70], and some models of colitis [74]. 

There are numerous methods available to study cell proliferation. The most reliable assay measures cell proliferation through the determination of DNA synthesis. The thymidine incorporation assay, as a “gold standard”, is the most commonly used assay [84]. Thymidine labeled with tritium (^3^H-thymidine) is incorporated into new strands of chromosomal DNA during the synthesis (S) phase of the cell cycle. Replication of DNA is a necessary step for subsequent cell division. 

The current study revealed that application of obestatin in animals without TNBS-induced colitis had no significant effect on mucosal DNA synthesis in the colon. This leads to the conclusion that, in animals with normal colonic mucous membranes, obestatin does not stimulate DNA synthesis, and thus, as it was previously noted, administration of this peptide appears safe, and neither poses a risk of hyperplasia or hypertrophy [74]. Additionally, administration of obestatin in rats with TNBS-induced colitis led to a considerable improvement of mucosal DNA synthesis in the colon, which indicated that in the course of TNBS-induced colitis, obestatin improved cell vitality and increased proliferation to some extent.

Adequate organ blood flow plays an extremely important role in maintaining mucosal integrity. Blood circulation supplies the colon with oxygen and HCO_3_^−^, removes H^+^ and CO_2_, and protects the mucosa from toxic agents diffusing from the lumen [85,86]. The experimental studies in rat models showed that exposure of various parts of the digestive tract, such as the oral cavity, esophagus, stomach, duodenum, and colon, to noxious factors caused no damage or minimal mucosal damage, so long as sufficient blood flow was maintained [55,57,87,88,89,90]. In this study, we observed that intrarectal administration of TNBS decreased mucosal blood flow in the colon. On the other hand, administration of obestatin significantly improved mucosal blood flow in the colon of animals with colitis. In contrast, administration of obestatin in rats without colitis had no effect on mucosal blood flow in the colon. These observations indicated that obestatin was involved in the improvement of colonic blood flow in rats with colitis, but this effect seemed to be an indirect influence of obestatin on colonic circulation. The improvement of colonic blood flow was probably related to the obestatin-evoked reduction in colonic damage and local inflammation. This observation was supported by previous studies showing that the relationship between mucosal blood flow and mucosal damage was bidirectional. An improvement of mucosal blood flow reduces mucosal damage and accelerates mucosal regeneration; there as, a reduction in mucosal blood flow intensifies mucosal damage [85,86].

White blood cells (WBCs), also called leukocytes, are the cells of the immune system. These cells participate in the protection of the body against infectious and toxic agents, as well as being involved in the inflammatory process [91,92]. On the basis of their appearance, WBCs are divided into granulocytes and agranulocytes. Granulocytes are then divided into neutrophils, eosinophils, and basophils; whereas agranulocytes include lymphocytes and monocytes. The number of leukocytes in the blood may vary in physiological and pathological conditions. Physiologically, WBC count rises after food intake, physical effort, stress, and emotional reactions. In pathological conditions, the main pathological causes of WBC-count elevation are infection and/or inflammation. Neutrophils are the first cells recruited to the colon, and their inflammatory response is believed to correspond with disease severity. 

Neutrophil-mediated inflammation can be a double-edged sword. In animal models, ablation of the neutrophil response had both favorable [93,94] and unfavorable consequences [95,96]. Neutrophil activity itself can lead to immune-mediated damage of host tissues, and therefore, for the best outcome of colitis therapy, a well-balanced and controlled neutrophil response is preferred. Neutrophils are also known to reflect the degree of inflammation [97]. Most clinical studies and scoring indices focus on total WBC count in colitis, and recent studies suggested that neutropenia should be regarded as a risk factor comparable to leukocytosis [98,99]. A decrease in WBC count debilitates the protective mechanism against infection and inflammation. In our presented study, WBC count reached a value of almost 8000 per mm^3^ of blood in control rats treated with saline without colitis. The administration of obestatin in the doses used had no significant effect on WBC count in rats without colitis. This observation suggested that doses of obestatin did not affect the immune system in this physiological condition. Colitis significantly increased WBC count, and the administration of obestatin in these rats reduced the level of this parameter. In the cases of obestatin given at doses of 8 or 16 nmol/kg, this effect was statistically significant. These observations indicated that the induction of colitis via TNBS enema led to the development of a systemic inflammatory response, and obestatin was able to reduce this effect. This systemic inflammation was, of course, induced by the local inflammatory process in the colon, and local inflammation still existed 14 days after induction. It was found upon macroscopic and microscopic examination of the colon, and manifested as changes in the mucosal colonic IL-1β concentration, and the activity of MPO.

IL-1β is a member of the IL-1 family, and plays a crucial role in the pathomechanism of inflammation [100,101,102]. It is mainly produced by activated macrophages in the form of proproteins, and is proteolytically processed into its active form by intracellular caspase 1 or extracellular neutrophil proteases. IL-1β is an important inflammatory mediator involved in a number of diverse cellular activities, such as cell proliferation, differentiation, and apoptosis [100,103,104]. The fundamental role of IL-1β depends on the activation of local and systemic pro-inflammatory responses, which lead to an acute phase of inflammation [101]. It acts directly and indirectly through the stimulation of the release of other pro-inflammatory factors, such as interleukin-6 (IL-6) and tumor necrosis factor α (TNFα) [105,106]. Our presented study showed that TNBS-induced colitis led to an increase in IL-1β concentration in the damaged tissue, and this effect was still observed 14 days after administration of TNBS. The administration of obestatin had no effect on mucosal concentration of IL-1β in rats without colitis. In rats with colitis, treatment with obestatin caused a significant decrease in the level of this pro-inflammatory factor in colonic mucosa.

Since myeloperoxidase (MPO) is released from the granules of neutrophils during inflammatory response, its activity corresponds with the degree of tissue infiltration by neutrophils [62,63,64]. In the presented study, induction of colitis with TNBS caused an increase in mucosal MPO activity, whereas obestatin treatment partially, but significantly, reversed this effect. This finding provided further evidence that obestatin exhibits anti-inflammatory effects in the colon. The above observation was consistent with our previous studies, which pointed to the protective effect of obestatin against acetic acid-induced colitis, and confirmed its healing properties in this model [74]. These data suggested that the anti-inflammatory effect of obestatin was independent of a primary course of colitis. 

The above findings on the inhibitory effect of obestatin on IL-1β levels, and MPO activity in colonic mucosa in rats with TNBS-induced colitis indicated that the effect of promoting healing was at least, in part, associated with the anti-inflammatory properties of obestatin. Moreover, the observation that obestatin did not affect mucosal IL-1β concentration, and MPO activity in the colon of rats without induced colitis suggested that obestatin did not disturb the immune system when no signs of inflammation were present.

As mentioned in the introduction, obestatin and ghrelin are products of the same gene, and are derived from a common preprohormone [1,2,3]. The protective and therapeutic effects of ghrelin were proven in various organs and experimental models [46,47,51,54,56,57,58,61,63,89]. Obestatin seemed to present similar effects on the inflammatory processes in the gut [67,68,69,70,74,75]. Our presented study showed that obestatin reduced the severity of TNBS-induced colitis in rats; however, the direct mechanism of its action remains unclear [1,7,8,15,16,17,18,19,20,21]. 

Inflammatory bowel disease (IBD) is a chronic and relapsing disease, and is an important clinical problem. IBD is characterized as the chronic dysregulation of the mucosal immune system in the gastrointestinal tract; however, its pathogenesis is still unclear. To investigate the mechanism, pathophysiology, and new methods of treatment of IBD, various animal models of experimental colitis were developed [78,80,107]. TNBS-induced models of colitis, typical for Crohn’s disease, result in inadequate cell-mediated immune response, and acute Th1 inflammation, which includes a dense colonic tissue infiltration by cluster of differentiation 4 (CD4) T cells, and the release of numerous potent pro-inflammatory agents. Clinical manifestations of acute colitis in animals include inconsistent stool formation, occult, or even bloody diarrhea, loss of body weight, piloerection of fur, decreased movements of the animals, and death [78].

Our current study showed that intraperitoneal administration of obestatin caused a reduction in the area of colonic damage, improved mucosal blood flow in the colon, and reduced white-blood-cell level, mucosal concentration of pro-inflammatory interleukin-1β, and mucosal activity of MPO, which accelerated the healing of TNBS-induced colitis. These protective and therapeutic effects of obestatin were dose-dependent. In conclusions, we can say that administration of obestatin reduces severity of TNBS-induced colitis, and was apparently associated with an obestatin-induced anti-inflammatory effect, accompanied by an improved local mucosal blood flow, and increased cell proliferation in colonic mucosa.

## 4. Materials and Methods

### 4.1. Animals and Treatment

Studies were conducted on eighty rats; their weights ranged from 220 to 250 g. The study complied with the experimental protocol approved by the First Local Committee of Ethics for the Care and Use of Laboratory Animals in Cracow (permit number 26/2016, released on 20 July 2016). During the experiments, animals were kept in cages stored at room temperature under a 12 h light–dark cycle. For 16 h prior to induction of colitis, the animals were fasted with free access to water. Then, water and food intake were available ad libitum.

The animals were randomly divided into eight experimental groups: (1) control rats without induction of colitis, and treated intraperitoneally (i.p.) with saline; (2) rats without induction of colitis, and treated i.p. with obestatin at a dose of 4 nmol/kg; (3) control rats without induction of colitis, and treated i.p. with obestatin at a dose of 8 nmol/kg; (4) control rats without induction of colitis, and treated i.p. with obestatin at a dose of 16 nmol/kg; (5) rats with colitis treated i.p. with saline; (6) rats with colitis treated i.p. with obestatin at a dose of 4 nmol/kg; (7) rats with colitis treated i.p. with obestatin at a dose of 8 nmol/kg; and (8) rats with colitis treated i.p. with obestatin at a dose of 16 nmol/kg.

Before induction of colitis, rats were fasted for 16 h, and anesthetized with ketamine (50 mg/kg i.p., Bioketan, Biowet, Gorzów Wielkopolski, Poland). The colitis was induced by intrarectal administration of 25 mg of 2,4,6-trinitrobenzene sulfonic acid (TNBS) dissolved in 0.25 mL of 50% ethanol [78,81]. Saline solution was similarly administered to rats without induction of colitis.

Starting 24 h before the rectal enema with saline or TNBS, the rats were treated with saline (groups 1 and 5) or obestatin (groups 2–4 and 6–8) administered i.p., twice per day for 4 consecutive days. Rat obestatin (Yanaihara Institute, Shizuoka, Japan) was given at doses of 4, 8, or 16 nmol/kg. These doses were chosen because previous studies showed that obestatin given at doses of 8 and 16 nmol/kg exhibited strong and repeatable therapeutic effects in the healing of acute pancreatitis, chronic gastric ulcers, and colitis [67,70,74].

Fourteen days after the induction of colitis, rats were again anesthetized with ketamine (50 mg/kg i.p., Bioketan, Vetoquinol Biowet, Gorzów Wielkopolski, Poland), and the severity of colitis was determined. 

### 4.2. Measurement of Colonic Blood Flow, Leukocytes in the Blood, and Colonic Damage

The abdominal cavity was opened, the colon was exposed, and the colonic blood flow was measured by means of a laser Doppler flowmeter (PeriFlux 4001Master monitor, Perimed AB, Järfälla, Sweden); the procedure was carried out in accordance with the previously described methodology [90]. The blood flow was measured at five points of the sigmoidal colon, and the main value of the records was expressed as a percentage of the value recorded in animals from the control group. 

After the measurement of colonic blood flow, arterial blood was taken from the abdominal aorta, and collected in tubes containing ethylenediaminetetraacetic acid (EDTA). The level of leukocytes (white blood cells—WBC) was determined using the automated hematology analyzer, Sysmex XE 2100 (Sysmex Corporation, Kobe, Japan).

After the measurement of colonic blood flow, and the collection of blood samples, animals were euthanized via exsanguination. Then, the area of mucosal damage in the colon was measured using a computerized planimeter (Morphomat, Carl Zeiss, Berlin, Germany), in accordance with the method previously described [55].

### 4.3. Biochemical Analysis

Following the measurements of colonic blood flow and the lesion area, histological examination of colonic samples, mucosal DNA synthesis (the index of mucosal proliferation), pro-inflammatory interleukin-1β concentration and myeloperoxidase activity was performed.

#### 4.3.1. Determination of DNA Synthesis in Colonic Mucosa

The role of DNA synthesis in colonic mucosa was determined via measurement of incorporated tritium-labeled thymidine into DNA, according to the methodology previously described [59,61,108]. Samples of colonic mucosa were incubated at 37 °C for 45 min in 2 mL of solution containing tritium-labeled thymidine ([^3^H]-thymidine, 20–30 Ci/mmol; Institute for Research, Production and Application of Radioisotopes, Prague, Czech Republic) with an activity of 8 μCi/mL. The level of incorporation of labeled thymidine into DNA was determined by measuring 0.5 mL DNA-containing supernatant in a liquid-scintillation system. The rate of DNA synthesis was expressed as the number of tritium-atom disintegrations per minute per microgram of DNA (dpm/μg DNA).

#### 4.3.2. Determination of Interleukin-1β Concentration in Colonic Mucosa

The colonic mucosa specimens were homogenized in phosphate buffer at a temperature of 4 °C. Next, the homogenate was centrifuged. The interleukin-1β concentration in the supernatant was determined using the Rat IL-1β Platinum ELISA kit (Bender MedSystem GmbH, Vienna, Austria). The value was expressed in ng per g of tissue.

#### 4.3.3. Determination of Myeloperoxidase Activity in Colonic Mucosa

Biopsy samples were homogenized in ice-cold potassium phosphate, and were stored at a temperature of −60 °C until the markings were conducted. Myeloperoxidase activity was assessed based on a modification of the method described by Bradley et al. [109]. Results, given in units per gram of tissue, were expressed as a percentage of the value recorded in the control group.

### 4.4. Histological Examination of the Colon

Samples of the colon were fixed in 10% buffered formaldehyde, and embedded in paraffin. Paraffin sections were stained with hematoxylin and eosin, and examined by the pathologist uninformed of the treatment implemented beforehand. The histological grading of colonic damage (i.e., ulceration, inflammation, depth of the lesion, and fibrosis) were determined using a scale devised by Vilaseca et al. [110], as previously described in detail [59]. For the histological grading of lesions, a scale ranging from 0 to 2 was used (0 = no lesions; 1 = small lesions < 3 mm; and 2 = large lesions > 3 mm). Inflammatory infiltration was graded from 0 to 3 (0 = none; 1 = small; 2 = moderate; and 3 = heavy), and the depth of lesions was assigned numbers from 0 to 3 (0 = no lesions; 1 = lesions reaching submucosa; 2 = lesions reaching muscularis propia; and 3 = lesions reaching serosa). For the evaluation of fibrosis, grades from 0 to 2 were applied (0 = none; 1 = mild; and 2 = severe). Moreover, the occurrence of inflammation of arterial vessels was assessed.

### 4.5. Statistical Analysis

Results were presented as mean values ± standard error of the mean (SEM). Analysis of variance was conducted, followed by Tukey’s multiple comparison test, using GraphPad Prism (GraphPad Software, San Diego, CA, USA). A *p*-value < 0.05 was considered statistically significant.

## Figures and Tables

**Table 1:** Influence of treatment with obestatin given intraperitoneally at doses of 4, 8, or 16 nmol/kg, and colitis evoked by rectal enema with trinitrobenzene sulfonic acid (TNBS) on morphological signs of colonic damage, observed 14 days after induction of colitis.

Groups	Morphological Changes			
	Grading of Colonic Damage (0–2)	Inflammatory Infiltration (0–3)	Depth of Damage (0–3)	Fibrosis (0–3)
Control (NaCl)	0	0	0	0
Obestatin 4	0	0	0	0
Obestatin 8	0	0	0	0
Obestatin 16	0	0	0	0
Colitis (TNBS) + NaCl	2	2	3	1
Colitis + Obestatin 4	1–2	1–2	1	2
Colitis + Obestatin 8	0–1	1	0–1	1
Colitis + Obestatin 16	0–1	1	0–1	1

Numbers represent the predominant histological grading in each group.

## References

[1] Zhang J.V., Ren P.-G., Avsian-Kretchmer O., Luo C.-W., Rauch R., Klein C., Hsueh A.J.W. (2005). Obestatin, a Peptide Encoded by the Ghrelin Gene, Opposes Ghrelin’s Effects on Food Intake. Science.

[2] Chen C.-Y., Asakawa A., Fujimiya M., Lee S.-D., Inui A. (2009). Ghrelin Gene Products and the Regulation of Food Intake and Gut Motility. Pharmacol. Rev..

[3] Ceranowicz P., Warzecha Z., Dembinski A. (2015). Peptidyl Hormones of Endocrine Cells Origin in the Gut—Their Discovery and Physiological Relevance. J. Physiol. Pharmacol..

[4] Furnes M.W., Stenstrom B., Tømmerås K., Skoglund T., Dickson S.L., Kulseng B., Zhao C.-M., Chen D. (2008). Feeding Behavior in Rats Subjected to Gastrectomy or Gastric Bypass Surgery. Eur. Surg. Res..

[5] Chanoine J.-P., Wong A.C.K., Barrios V. (2006). Obestatin, Acylated and Total Ghrelin Concentrations in the Perinatal Rat Pancreas. Horm. Res..

[6] Bang A.S., Soule S.G., Yandle T.G., Richards A.M., Pemberton C.J. (2007). Characterisation of Proghrelin Peptides in Mammalian Tissue and Plasma. J. Endocrinol..

[7] Granata R., Baragli A., Settanni F., Scarlatti F., Ghigo E. (2010). Unraveling the Role of the Ghrelin Gene Peptides in the Endocrine Pancreas. J. Mol. Endocrinol..

[8] Granata R., Settanni F., Gallo D., Trovato L., Biancone L., Cantaluppi V., Nano R., Annunziata M., Campiglia P., Arnoletti E. (2008). Obestatin Promotes Survival of Pancreatic Beta-Cells and Human Islets and Induces Expression of Genes Involved in the Regulation of Beta-Cell Mass and Function. Diabetes.

[9] Dun S.L., Brailoiu G.C., Brailoiu E., Yang J., Chang J.K., Dun N.J. (2006). Distribution and Biological Activity of Obestatin in the Rat. J. Endocrinol..

[10] Grönberg M., Tsolakis A.V., Magnusson L., Janson E.T., Saras J. (2008). Distribution of Obestatin and Ghrelin in Human Tissues: Immunoreactive Cells in the Gastrointestinal Tract, Pancreas, and Mammary Glands. J. Histochem. Cytochem. Off. J. Histochem. Soc..

[11] Volante M., Rosas R., Ceppi P., Rapa I., Cassoni P., Wiedenmann B., Settanni F., Granata R., Papotti M. (2009). Obestatin in Human Neuroendocrine Tissues and Tumours: Expression and Effect on Tumour Growth. J. Pathol..

[12] Zhao C.-M., Furnes M.W., Stenström B., Kulseng B., Chen D. (2008). Characterization of Obestatin- and Ghrelin-Producing Cells in the Gastrointestinal Tract and Pancreas of Rats: An Immunohistochemical and Electron-Microscopic Study. Cell Tissue Res..

[13] Gurriarán-Rodríguez U., Santos-Zas I., Al-Massadi O., Mosteiro C.S., Beiroa D., Nogueiras R., Crujeiras A.B., Seoane L.M., Señarís J., García-Caballero T. (2012). The Obestatin/GPR39 System Is up-Regulated by Muscle Injury and Functions as an Autocrine Regenerative System. J. Biol. Chem..

[14] Moretti E., Vindigni C., Tripodi S.A., Mazzi L., Nuti R., Figura N., Collodel G. (2014). Immunolocalisation of Ghrelin and Obestatin in Human Testis, Seminal Vesicles, Prostate and Spermatozoa. Andrologia.

[15] Zhang J.V., Jahr H., Luo C.-W., Klein C., Van Kolen K., Ver Donck L., De A., Baart E., Li J., Moechars D. (2008). Obestatin Induction of Early-Response Gene Expression in Gastrointestinal and Adipose Tissues and the Mediatory Role of G Protein-Coupled Receptor, GPR39. Mol. Endocrinol..

[16] Zhang J.V., Li L., Huang Q., Ren P.-G. (2013). Obestatin Receptor in Energy Homeostasis and Obesity Pathogenesis. Prog. Mol. Biol. Transl. Sci..

[17] Ren G., He Z., Cong P., Chen H., Guo Y., Yu J., Liu Z., Ji Q., Song Z., Chen Y. (2013). Peripheral Administration of TAT-Obestatin Can Influence the Expression of Liporegulatory Genes but Fails to Affect Food Intake in Mice. Peptides.

[18] Dong X.-Y., He J.-M., Tang S.-Q., Li H.-Y., Jiang Q.-Y., Zou X.-T. (2009). Is GPR39 the Natural Receptor of Obestatin?. Peptides.

[19] Chartrel N., Alvear-Perez R., Leprince J., Iturrioz X., Reaux-Le Goazigo A., Audinot V., Chomarat P., Coge F., Nosjean O., Rodriguez M. (2007). Comment on “Obestatin, a Peptide Encoded by the Ghrelin Gene, Opposes Ghrelin’s Effects on Food Intake”. Science.

[20] Lauwers E., Landuyt B., Arckens L., Schoofs L., Luyten W. (2006). Obestatin Does Not Activate Orphan G Protein-Coupled Receptor GPR39. Biochem. Biophys. Res. Commun..

[21] Granata R., Gallo D., Luque R.M., Baragli A., Scarlatti F., Grande C., Gesmundo I., Córdoba-Chacón J., Bergandi L., Settanni F. (2012). Obestatin Regulates Adipocyte Function and Protects against Diet-Induced Insulin Resistance and Inflammation. FASEB J..

[22] Alloatti G., Arnoletti E., Bassino E., Penna C., Perrelli M.G., Ghé C., Muccioli G. (2010). Obestatin Affords Cardioprotection to the Ischemic-Reperfused Isolated Rat Heart and Inhibits Apoptosis in Cultures of Similarly Stressed Cardiomyocytes. Am. J. Physiol. Heart Circ. Physiol..

[23] Zhang Q., Dong X.-W., Xia J.-Y., Xu K.-Y., Xu Z.-R. (2017). Obestatin Plays Beneficial Role in Cardiomyocyte Injury Induced by Ischemia-Reperfusion in Vivo and in Vitro. Med. Sci. Monit..

[24] Penna C., Tullio F., Femminò S., Rocca C., Angelone T., Cerra M.C., Gallo M.P., Gesmundo I., Fanciulli A., Brizzi M.F. (2017). Obestatin Regulates Cardiovascular Function and Promotes Cardioprotection through the Nitric Oxide Pathway. J. Cell. Mol. Med..

[25] Wang J., Wang L., Wei L., Wu J., Wei N., Kong X., Tian Z. (2010). Association of gastric emptying with ghrelin, obestatin and receptor (GHSR, GPR-39) in hypothalamus of diabetic rats. Zhonghua Yi Xue Za Zhi.

[26] Ataka K., Inui A., Asakawa A., Kato I., Fujimiya M. (2008). Obestatin Inhibits Motor Activity in the Antrum and Duodenum in the Fed State of Conscious Rats. Am. J. Physiol. Gastrointest. Liver Physiol..

[27] Fujimiya M., Asakawa A., Ataka K., Kato I., Inui A. (2008). Different Effects of Ghrelin, Des-Acyl Ghrelin and Obestatin on Gastroduodenal Motility in Conscious Rats. World J. Gastroenterol. WJG.

[28] Qader S.S., Håkanson R., Rehfeld J.F., Lundquist I., Salehi A. (2008). Proghrelin-Derived Peptides Influence the Secretion of Insulin, Glucagon, Pancreatic Polypeptide and Somatostatin: A Study on Isolated Islets from Mouse and Rat Pancreas. Regul. Pept..

[29] Lippl F., Erdmann J., Lichter N., Tholl S., Wagenpfeil S., Adam O., Schusdziarra V. (2008). Relation of Plasma Obestatin Levels to BMI, Gender, Age and Insulin. Horm. Metab. Res..

[30] Shao L., Zhao Y.-T., Teng L.-L., Li M.-Z., Jiang H. (2014). Circulating Obestatin Levels Correlate with Fasting Insulin and HOMA-IR but Not with Hypertension in Elderly Men. Cell Biochem. Biophys..

[31] Ren A.-J., Guo Z.-F., Wang Y.-K., Wang L.-G., Wang W.-Z., Lin L., Zheng X., Yuan W.-J. (2008). Inhibitory Effect of Obestatin on Glucose-Induced Insulin Secretion in Rats. Biochem. Biophys. Res. Commun..

[32] Şen L.S., Karakoyun B., Yeğen C., Akkiprik M., Yüksel M., Ercan F., Özer A., Yeğen B.Ç. (2015). Treatment with Either Obestatin or Ghrelin Attenuates Mesenteric Ischemia-Reperfusion-Induced Oxidative Injury of the Ileum and the Remote Organ Lung. Peptides.

[33] Khirazova E.E., Golubeva M.G., Maslova M.V., Graf A.V., Maklakova A.S., Baizhumanov A.A., Trofimova L.K., Sokolova N.A., Kamenskii A.A. (2013). Effect of Anorexigenic Peptide Obestatin on Platelet Aggregation and Osmotic Resistance of Erythrocytes. Bull. Exp. Biol. Med..

[34] Liu Y., Xing Y.-X., Gao X.-Y., Kuang H.-Y., Zhang J., Liu R. (2018). Obestatin Prevents H_2_O_2_-Induced Damage through Activation of TrkB in RGC-5 Cells. Biomed. Pharmacother..

[35] Słupecka M., Woliński J., Herman A.P., Ochniewicz P., Kornacka M.K. (2012). Biological role of obestatin in physiology and pathophysiology. Med. Wieku Rozw..

[36] Trovato L., Gallo D., Settanni F., Gesmundo I., Ghigo E., Granata R. (2014). Obestatin: Is It Really Doing Something?. Front. Horm. Res..

[37] Xing Y.-X., Yang L., Kuang H.-Y., Gao X.-Y., Liu H.-L. (2017). Function of Obestatin in the Digestive System. Nutrition.

[38] Ren A.-J., Guo Z.-F., Wang Y.-K., Lin L., Zheng X., Yuan W.-J. (2009). Obestatin, Obesity and Diabetes. Peptides.

[39] Zhang N., Yuan C., Li Z., Li J., Li X., Li C., Li R., Wang S.-R. (2011). Meta-Analysis of the Relationship between Obestatin and Ghrelin Levels and the Ghrelin/Obestatin Ratio with Respect to Obesity. Am. J. Med. Sci..

[40] Zou C.C., Liang L., Wang C.L., Fu J.F., Zhao Z.Y. (2009). The Change in Ghrelin and Obestatin Levels in Obese Children after Weight Reduction. Acta Paediatr..

[41] Wali P., King J., He Z., Tonb D., Horvath K. (2014). Ghrelin and Obestatin Levels in Children with Failure to Thrive and Obesity. J. Pediatr. Gastroenterol. Nutr..

[42] Harsch I.A., Koebnick C., Tasi A.M., Hahn E.G., Konturek P.C. (2009). Ghrelin and Obestatin Levels in Type 2 Diabetic Patients with and without Delayed Gastric Emptying. Dig. Dis. Sci..

[43] Qi X., Li L., Yang G., Liu J., Li K., Tang Y., Liou H., Boden G. (2007). Circulating Obestatin Levels in Normal Subjects and in Patients with Impaired Glucose Regulation and Type 2 Diabetes Mellitus. Clin. Endocrinol..

[44] Butler M.G., Bittel D.C. (2007). Plasma Obestatin and Ghrelin Levels in Subjects with Prader-Willi Syndrome. Am. J. Med. Genet..

[45] Liu W., Yue H., Zhang J., Pu J., Yu Q. (2014). Effects of Plasma Ghrelin, Obestatin, and Ghrelin/Obestatin Ratio on Blood Pressure Circadian Rhythms in Patients with Obstructive Sleep Apnea Syndrome. Chin. Med. J..

[46] Sibilia V., Rindi G., Pagani F., Rapetti D., Locatelli V., Torsello A., Campanini N., Deghenghi R., Netti C. (2003). Ghrelin Protects against Ethanol-Induced Gastric Ulcers in Rats: Studies on the Mechanisms of Action. Endocrinology.

[47] Brzozowski T., Konturek P.C., Konturek S.J., Kwiecień S., Drozdowicz D., Bielanski W., Pajdo R., Ptak A., Nikiforuk A., Pawlik W.W. (2004). Exogenous and Endogenous Ghrelin in Gastroprotection against Stress-Induced Gastric Damage. Regul. Pept..

[48] Konturek P.C., Brzozowski T., Walter B., Burnat G., Hess T., Hahn E.G., Konturek S.J. (2006). Ghrelin-Induced Gastroprotection against Ischemia-Reperfusion Injury Involves an Activation of Sensory Afferent Nerves and Hyperemia Mediated by Nitric Oxide. Eur. J. Pharmacol..

[49] Brzozowski T., Konturek P.C., Sliwowski Z., Drozdowicz D., Kwiecien S., Pawlik M., Pajdo R., Konturek S.J., Pawlik W.W., Hahn E.G. (2006). Neural Aspects of Ghrelin-Induced Gastroprotection against Mucosal Injury Induced by Noxious Agents. J. Physiol. Pharmacol..

[50] Işeri S.O., Sener G., Yüksel M., Contuk G., Cetinel S., Gedik N., Yegen B.C. (2005). Ghrelin against Alendronate-Induced Gastric Damage in Rats. J. Endocrinol..

[51] Dembinski A., Warzecha Z., Ceranowicz P., Tomaszewska R., Stachura J., Konturek S.J., Konturek P.C. (2003). Ghrelin Attenuates the Development of Acute Pancreatitis in Rat. J. Physiol. Pharmacol..

[52] Dembiński A., Warzecha Z., Ceranowicz P., Cieszkowski J., Pawlik W.W., Tomaszewska R., Kuśnierz-Cabala B., Naskalski J.W., Kuwahara A., Kato I. (2006). Role of Growth Hormone and Insulin-like Growth Factor-1 in the Protective Effect of Ghrelin in Ischemia/Reperfusion-Induced Acute Pancreatitis. Growth Horm. IGF Res..

[53] Bonior J., Warzecha Z., Ceranowicz P., Gajdosz R., Pierzchalski P., Kot M., Leja-Szpak A., Nawrot-Porąbka K., Link-Lenczowski P., Pędziwiatr M. (2017). Capsaicin-Sensitive Sensory Nerves Are Necessary for the Protective Effect of Ghrelin in Cerulein-Induced Acute Pancreatitis in Rats. Int. J. Mol. Sci..

[54] Warzecha Z., Dembinski A. (2012). Protective and Therapeutic Effects of Ghrelin in the Gut. Curr. Med. Chem..

[55] Warzecha Z., Kownacki P., Ceranowicz P., Dembinski M., Cieszkowski J., Dembinski A. (2013). Ghrelin Accelerates the Healing of Oral Ulcers in Non-Sialoadenectomized and Sialoadenectomized Rats. J. Physiol. Pharmacol..

[56] Cieszkowski J., Warzecha Z., Ceranowicz P., Ceranowicz D., Kusnierz-Cabala B., Pedziwiatr M., Dembinski M., Ambrozy T., Kaczmarzyk T., Pihut M. (2017). Therapeutic Effect of Exogenous Ghrelin in the Healing of Gingival Ulcers Is Mediated by the Release of Endogenous Growth Hormone and Insulin-like Growth Factor-1. J. Physiol. Pharmacol..

[57] Ceranowicz P., Warzecha Z., Dembinski A., Sendur R., Cieszkowski J., Ceranowicz D., Pawlik W.W., Kuwahara A., Kato I., Konturek P.C. (2009). Treatment with Ghrelin Accelerates the Healing of Acetic Acid-Induced Gastric and Duodenal Ulcers in Rats. J. Physiol. Pharmacol. Off. J. Pol. Physiol. Soc..

[58] Konturek P.C., Brzozowski T., Engel M., Burnat G., Gaca P., Kwiecien S., Pajdo R., Konturek S.J. (2009). Ghrelin Ameliorates Colonic Inflammation. Role of Nitric Oxide and Sensory Nerves. J. Physiol. Pharmacol..

[59] Maduzia D., Matuszyk A., Ceranowicz D., Warzecha Z., Ceranowicz P., Fyderek K., Galazka K., Dembinski A. (2015). The Influence of Pretreatment with Ghrelin on the Development of Acetic-Acid-Induced Colitis in Rats. J. Physiol. Pharmacol..

[60] Matuszyk A., Ceranowicz D., Warzecha Z., Ceranowicz P., Fyderek K., Gałązka K., Cieszkowski J., Bonior J., Jaworek J., Pihut M. (2015). The Influence of Ghrelin on the Development of Dextran Sodium Sulfate-Induced Colitis in Rats. BioMed Res. Int..

[61] Matuszyk A., Ceranowicz P., Warzecha Z., Cieszkowski J., Ceranowicz D., Gałązka K., Bonior J., Jaworek J., Bartuś K., Gil K. (2016). Exogenous Ghrelin Accelerates the Healing of Acetic Acid-Induced Colitis in Rats. Int. J. Mol. Sci..

[62] Ceranowicz P., Warzecha Z., Cieszkowski J., Ceranowicz D., Kuśnierz-Cabala B., Bonior J., Jaworek J., Ambroży T., Gil K., Olszanecki R. (2017). Essential Role of Growth Hormone and IGF-1 in Therapeutic Effect of Ghrelin in the Course of Acetic Acid-Induced Colitis. Int. J. Mol. Sci..

[63] Warzecha Z., Ceranowicz P., Dembinski A., Cieszkowski J., Kusnierz-Cabala B., Tomaszewska R., Kuwahara A., Kato I. (2010). Therapeutic Effect of Ghrelin in the Course of Cerulein-Induced Acute Pancreatitis in Rats. J. Physiol. Pharmacol..

[64] Ceranowicz D., Warzecha Z., Dembinski A., Ceranowicz P., Cieszkowski J., Kusnierz-Cabala B., Tomaszewska R., Kuwahara A., Kato I. (2010). Role of Hormonal Axis, Growth Hormone—IGF-1, in the Therapeutic Effect of Ghrelin in the Course of Cerulein-Induced Acute Pancreatitis. J. Physiol. Pharmacol..

[65] Bukowczan J., Warzecha Z., Ceranowicz P., Kusnierz-Cabala B., Tomaszewska R., Dembinski A. (2015). Therapeutic Effect of Ghrelin in the Course of Ischemia/Reperfusion-Induced Acute Pancreatitis. Curr. Pharm. Des..

[66] Bonior J., Ceranowicz P., Gajdosz R., Kuśnierz-Cabala B., Pierzchalski P., Warzecha Z., Dembiński A., Pędziwiatr M., Kot M., Leja-Szpak A. (2017). Molecular Ghrelin System in the Pancreatic Acinar Cells: The Role of the Polypeptide, Caerulein and Sensory Nerves. Int. J. Mol. Sci..

[67] Ceranowicz P., Warzecha Z., Dembinski A., Cieszkowski J., Dembinski M., Sendur R., Kusnierz-Cabala B., Tomaszewska R., Kuwahara A., Kato I. (2009). Pretreatment with Obestatin Inhibits the Development of Cerulein-Induced Pancreatitis. J. Physiol. Pharmacol..

[68] Bukowczan J., Warzecha Z., Ceranowicz P., Kuśnierz-Cabala B., Tomaszewska R., Dembinski A. (2015). Pretreatment with Obestatin Reduces the Severity of Ischemia/Reperfusion-Induced Acute Pancreatitis in Rats. Eur. J. Pharmacol..

[69] Bukowczan J., Warzecha Z., Ceranowicz P., Kuśnierz-Cabala B., Tomaszewska R. (2015). Obestatin Accelerates the Recovery in the Course of Ischemia/Reperfusion-Induced Acute Pancreatitis in Rats. PLoS ONE.

[70] Dembiński A., Warzecha Z., Ceranowicz P., Cieszkowski J., Dembiński M., Ptak-Belowska A., Kuwahara A., Kato I. (2011). Administration of Obestatin Accelerates the Healing of Chronic Gastric Ulcers in Rats. Med. Sci. Monit. Int. Med..

[71] Alexandridis E., Zisimopoulos A., Liratzopoulos N., Katsos I., Manolas K., Kouklakis G. (2009). Obestatin/Ghrelin Ratio: A New Activity Index in Inflammatory Bowel Diseases. Inflamm. Bowel Dis..

[72] Jung J.Y., Jeong J.B., Kim J.W., Kim S.H., Koh S.-J., Kim B.G., Lee K.L. (2015). Circulating Ghrelin Levels and Obestatin/Ghrelin Ratio as a Marker of Activity in Ulcerative Colitis. Intest. Res..

[73] Russo F., Chimienti G., Linsalata M., Clemente C., Orlando A., Riezzo G. (2017). The Obestatin/Ghrelin Ratio and Ghrelin Genetics in Adult Celiac Patients before and after a Gluten-Free Diet, in Irritable Bowel Syndrome Patients and Healthy Individuals. Eur. J. Gastroenterol. Hepatol..

[74] Matuszyk A., Ceranowicz P., Warzecha Z., Cieszkowski J., Bonior J., Jaworek J., Kuśnierz-Cabala B., Konturek P., Ambroży T., Dembiński A. (2016). Obestatin Accelerates the Healing of Acetic Acid-Induced Colitis in Rats. Oxid. Med. Cell. Longev..

[75] Pamukcu O., Kumral Z.N.O., Ercan F., Yegen B.C., Ertem D. (2013). Anti-Inflammatory Effect of Obestatin and Ghrelin in Dextran Sulfate Sodium-Induced Colitis in Rats. J. Pediatr. Gastroenterol. Nutr..

[76] Brenna Ø., Furnes M.W., Drozdov I., van Beelen Granlund A., Flatberg A., Sandvik A.K., Zwiggelaar R.T.M., Mårvik R., Nordrum I.S., Kidd M. (2013). Relevance of TNBS-Colitis in Rats: A Methodological Study with Endoscopic, Histologic and Transcriptomic Characterization and Correlation to IBD. PLoS ONE.

[77] Te Velde A.A., de Kort F., Sterrenburg E., Pronk I., ten Kate F.J.W., Hommes D.W., van Deventer S.J.H. (2007). Comparative Analysis of Colonic Gene Expression of Three Experimental Colitis Models Mimicking Inflammatory Bowel Disease. Inflamm. Bowel Dis..

[78] Antoniou E., Margonis G.A., Angelou A., Pikouli A., Argiri P., Karavokyros I., Papalois A., Pikoulis E. (2016). The TNBS-Induced Colitis Animal Model: An Overview. Ann. Med. Surg..

[79] Neurath M.F., Fuss I., Kelsall B.L., Stüber E., Strober W. (1995). Antibodies to Interleukin 12 Abrogate Established Experimental Colitis in Mice. J. Exp. Med..

[80] Kawada M., Arihiro A., Mizoguchi E. (2007). Insights from Advances in Research of Chemically Induced Experimental Models of Human Inflammatory Bowel Disease. World J. Gastroenterol..

[81] Morris G.P., Beck P.L., Herridge M.S., Depew W.T., Szewczuk M.R., Wallace J.L. (1989). Hapten-Induced Model of Chronic Inflammation and Ulceration in the Rat Colon. Gastroenterology.

[82] Elson C.O., Beagley K.W., Sharmanov A.T., Fujihashi K., Kiyono H., Tennyson G.S., Cong Y., Black C.A., Ridwan B.W., McGhee J.R. (1996). Hapten-Induced Model of Murine Inflammatory Bowel Disease: Mucosa Immune Responses and Protection by Tolerance. J. Immunol..

[83] Fitzpatrick L.R., Meirelles K., Small J.S., Puleo F.J., Koltun W.A., Cooney R.N. (2010). A New Model of Chronic Hapten-Induced Colitis in Young Rats. J. Pediatr. Gastroenterol. Nutr..

[84] Flaxman B.A., Harper R.A. (1975). Primary Cell Culture for Biochemical Studies of Human Keratinocytes. A Method for Production of Very Large Numbers of Cells without the Necessity of Subculturing Techniques. Br. J. Dermatol..

[85] Vigneri S., Scialabba A., Termini R., Germanà B., Vianello F., Grassi S.A., Plebani M., Di Mario F. (1992). Pathophysiology of the Gastric Microcirculation. Ital. J. Gastroenterol..

[86] Sørbye H., Svanes K. (1994). The Role of Blood Flow in Gastric Mucosal Defence, Damage and Healing. Dig. Dis..

[87] Orlando R.C. (2010). The Integrity of the Esophageal Mucosa. Balance between Offensive and Defensive Mechanisms. Best Pract. Res. Clin. Gastroenterol..

[88] Abdel-Salam O.M., Czimmer J., Debreceni A., Szolcsányi J., Mózsik G. (2001). Gastric Mucosal Integrity: Gastric Mucosal Blood Flow and Microcirculation. An Overview. J. Physiol. Paris.

[89] Warzecha Z., Ceranowicz D., Dembiński A., Ceranowicz P., Cieszkowski J., Kuwahara A., Kato I., Dembiński M., Konturek P.C. (2012). Ghrelin Accelerates the Healing of Cysteamine-Induced Duodenal Ulcers in Rats. Med. Sci. Monit..

[90] Leung F.W., Su K.C., Pique J.M., Thiefin G., Passaro E., Guth P.H. (1992). Superior Mesenteric Artery Is More Important than Inferior Mesenteric Artery in Maintaining Colonic Mucosal Perfusion and Integrity in Rats. Dig. Dis. Sci..

[91] Osborn L. (1990). Leukocyte Adhesion to Endothelium in Inflammation. Cell.

[92] Rich R., Fleisher T., Shearer W., Schroeder H., Frew A., Weyand C. (2012). Clinical Immunology: Principles and Practice.

[93] Conlan J.W., North R.J. (1994). Neutrophils Are Essential for Early Anti-Listeria Defense in the Liver, but Not in the Spleen or Peritoneal Cavity, as Revealed by a Granulocyte-Depleting Monoclonal Antibody. J. Exp. Med..

[94] Daley J.M., Thomay A.A., Connolly M.D., Reichner J.S., Albina J.E. (2008). Use of Ly6G-Specific Monoclonal Antibody to Deplete Neutrophils in Mice. J. Leukoc. Biol..

[95] Reber L.L., Gillis C.M., Starkl P., Jönsson F., Sibilano R., Marichal T., Gaudenzio N., Bérard M., Rogalla S., Contag C.H. (2017). Neutrophil Myeloperoxidase Diminishes the Toxic Effects and Mortality Induced by Lipopolysaccharide. J. Exp. Med..

[96] Hock H., Hamblen M.J., Rooke H.M., Traver D., Bronson R.T., Cameron S., Orkin S.H. (2003). Intrinsic Requirement for Zinc Finger Transcription Factor Gfi-1 in Neutrophil Differentiation. Immunity.

[97] Sugimoto M.A., Sousa L.P., Pinho V., Perretti M., Teixeira M.M. (2016). Resolution of Inflammation: What Controls Its Onset?. Front. Immunol..

[98] Sebastian S., Ashton K., Houston Y., Diggory T.M., Dore P. (2012). Anti-TNF Therapy Induced Immune Neutropenia in Crohns Disease-Report of 2 Cases and Review of Literature. J. Crohn’s Colitis.

[99] Rodrigues F.G., Dasilva G., Wexner S.D. (2017). Neutropenic Enterocolitis. World J. Gastroenterol..

[100] Dinarello C.A., Wolff S.M. (1993). The Role of Interleukin-1 in Disease. N. Engl. J. Med..

[101] Dinarello C.A. (2009). Immunological and Inflammatory Functions of the Interleukin-1 Family. Annu. Rev. Immunol..

[102] Garlanda C., Dinarello C.A., Mantovani A. (2013). The Interleukin-1 Family: Back to the Future. Immunity.

[103] Lopez-Castejon G., Brough D. (2011). Understanding the Mechanism of IL-1β Secretion. Cytokine Growth Factor Rev..

[104] Hogquist K.A., Nett M.A., Unanue E.R., Chaplin D.D. (1991). Interleukin 1 Is Processed and Released during Apoptosis. Proc. Natl. Acad. Sci. USA.

[105] Dinarello C.A. (2011). A Clinical Perspective of IL-1β as the Gatekeeper of Inflammation. Eur. J. Immunol..

[106] Dinarello C.A., Simon A., van der Meer J.W.M. (2012). Treating Inflammation by Blocking Interleukin-1 in a Broad Spectrum of Diseases. Nat. Rev. Drug Discov..

[107] Randhawa P.K., Singh K., Singh N., Jaggi A.S. (2014). A Review on Chemical-Induced Inflammatory Bowel Disease Models in Rodents. Korean J. Physiol. Pharmacol..

[108] Warzecha Z., Dembiński A., Brzozowski T., Ceranowicz P., Pajdo R., Niemiec J., Drozdowicz D., Mitis-Musioł M., Konturek S.J. (2000). Gastroprotective Effect of Histamine and Acid Secretion on Ammonia-Induced Gastric Lesions in Rats. Scand. J. Gastroenterol..

[109] Bradley P.P., Priebat D.A., Christensen R.D., Rothstein G. (1982). Measurement of Cutaneous Inflammation: Estimation of Neutrophil Content with an Enzyme Marker. J. Investig. Dermatol..

[110] Vilaseca J., Salas A., Guarner F., Rodríguez R., Martínez M., Malagelada J.R. (1990). Dietary Fish Oil Reduces Progression of Chronic Inflammatory Lesions in a Rat Model of Granulomatous Colitis. Gut.

